# Association between fear of cancer recurrence and emotional distress in breast cancer: a latent profile and moderation analysis

**DOI:** 10.3389/fpsyt.2025.1521555

**Published:** 2025-03-27

**Authors:** Yingting Jiang, Hongman Li, Ying Xiong, Xiaoting Zheng, Yanjun Liu, Jian Zhou, Zengjie Ye

**Affiliations:** ^1^ School of Nursing, Guangzhou University of Chinese Medicine, Guangzhou, Guangdong, China; ^2^ The First Affiliated Hospital of Guangzhou University of Traditional Chinese Medicine, Guangzhou, Guangdong, China; ^3^ School of Nursing, Guangzhou Medical University, Guangzhou, Guangdong, China

**Keywords:** fear of cancer recurrence, resilience, emotional distress, latent profile analysis, moderation analysis

## Abstract

**Background:**

Breast cancer patients often experience significant psychological challenges, particularly fear of cancer recurrence (FCR), which is a prevalent and distressing concern following diagnosis. FCR can lead to heightened emotional distress, including anxiety and depression. Resilience, the ability to adapt positively to adversity, may play a crucial role in mitigating these negative emotional outcomes. This study aims to explore the heterogeneity of FCR among breast cancer patients and examine the moderating effect of resilience on the relationship between FCR and emotional distress.

**Materials and methods:**

A cohort of 398 breast cancer patients participated in the Be Resilient to Breast Cancer (BRBC) program between May and December 2023. Surveys were administered to assess FCR, resilience, and emotional distress levels. Data were analyzed using two approaches: latent profile analysis (LPA) to identify distinct FCR profiles and moderation analysis to evaluate the role of resilience.

**Results:**

Three distinct FCR profiles were identified: low (27.5%), middle (53%), and high (19.5%). Resilience significantly moderated the association between FCR and anxiety (B = 0.115, SE = 0.046, *P* = 0.014), but no significant moderating effect was observed for depression.

**Discussion:**

The findings highlight significant heterogeneity in FCR among breast cancer patients, with a substantial proportion experiencing moderate to high levels of FCR. Resilience was found to buffer the impact of FCR on anxiety, suggesting that interventions aimed at enhancing resilience could alleviate anxiety related to FCR in this population. These results underscore the importance of incorporating resilience-focused strategies into psychological therapies for breast cancer patients.

## 1 Introduction

Breast cancer is the most prevalent cancer among women globally, with 2.3 million new cases annually, accounting for 24.5% of all female cancers (GLOBOCAN 2022) (1). Risk factors include genetic (e.g., BRCA1/2 mutations), hormonal (e.g., prolonged estrogen exposure), and lifestyle factors (e.g., obesity, physical inactivity) (2, 3). Despite advancements in screening and treatment that have improved 5-year survival rates, particularly in high-income countries, breast cancer remains a leading cause of cancer-related mortality worldwide. This is especially true in low- and middle-income countries (LMICs), where limited access to early detection and treatment contributes to higher mortality rates (4, 5). Disparities in healthcare resources and infrastructure further exacerbate these challenges, with LMICs bearing a disproportionate burden of breast cancer-related deaths (6).

Patients diagnosed with breast cancer frequently experience significant emotional distress, predominantly manifesting as anxiety and depression (7). These psychological challenges persist from diagnosis through treatment completion (8) and adversely affect treatment adherence, compromise outcomes, and reduce quality of life (9, 10). Additionally, these emotional distresses are bound with an increased hazard of condition deterioration and metastasis, ultimately contributing to lower survival rates (11, 12). In addition to anxiety and depression, fear of cancer recurrence (FCR) is another significant psychological challenge faced by breast cancer patients (13). FCR is characterized by persistent preoccupation with physical symptoms (14), avoidance of medical tests or excessive use of medical resources (15), and heightened anxiety and depression. Young female breast cancer patients are particularly vulnerable to FCR (16), with incidence rates exceeding 60% in some studies (17, 18). FCR significantly impacts both treatment and daily life. In treatment, it may lead to non-adherence to follow-up schedules or excessive use of healthcare resources (13, 19). It can also exacerbate psychological distress, reducing patients’ ability to cope with treatment side effects and overall quality of life (20). In daily life, FCR often impairs social functioning, disrupts sleep patterns, and reduces engagement in physical activities, further diminishing well-being (21).

Resilience constitutes a fundamental aspect of positive psychology, defined as an individual’s capacity to adapt themselves when facing damage, misfortune and gross life stressors (22). It counteracts the pessimistic consequences of stress, reduces the incidence of anxiety, depression, and FCR, while being positively correlated with enhanced well-being and mental health. Research indicates that the resilience exhibited by patients following carcinoma operation is conversely correlated with the severity of FCR (23). In breast cancer patients specifically, studies have shown that higher resilience levels are associated with lower FCR and better emotional adjustment (24). What’s more, individuals who possess greater psychosocial resources tend to experience a weaker correlation between stress and depressive symptoms (25). Additionally, prior investigations have established a positive connection between FCR and emotional distress (26, 27). Nonetheless, few empirical studies have explored the moderating effect of resilience on the relationship between FCR and emotional distress in breast cancer patients. This study seeks to provide new insights into how resilience influences the interplay between FCR and emotional distress in breast cancer patients. To address this shortcoming, the primary objectives of this study are twofold: (1) to explore the heterogeneity of FCR among breast cancer patients, and (2) to validate the moderating role of resilience in the relationship between FCR and emotional distress, particularly focusing on anxiety and depression.

## 2 Theoretical framework

FCR is a significant psychological challenge experienced by breast cancer patients, characterized by persistent and excessive worry about the potential return or progression of cancer (28). Patients with high levels of FCR often exhibit negative coping behaviors, such as avoidance or excessive medical reassurance-seeking, which exacerbate emotional distress and reduce treatment adherence (29). This phenomenon not only impairs patients’ ability to manage their illness but also undermines their confidence in recovery, ultimately hindering the healing process (30). According to Richard S. Lazarus’ Stress and Coping Theory, individuals’ emotional responses to stressors depend on their cognitive appraisal and coping strategies (31, 32). In the context of breast cancer, FCR serves as a significant stressor, and patients’ cognitive appraisal of their condition (e.g., viewing recurrence as catastrophic) can intensify anxiety and depression, creating a vicious cycle of emotional distress (33, 34). Based on this theoretical foundation, we propose H1: FCR is positively correlated with emotional distress in breast cancer patients.

FCR is a multifaceted construct that manifests differently across individuals (35). A qualitative study revealed that patients experiencing FCR often exhibit preoccupation with mortality, feelings of isolation, intolerance for uncertainty, and persistent cancer-related thoughts that disrupt daily functioning (34). Stress and Coping Theory emphasizes the importance of understanding individual differences in cognitive appraisal and coping responses to stressors (31). Latent Profile Analysis (LPA) provides a person-centered approach to identify distinct subgroups based on FCR patterns, enabling the characterization of high-risk subpopulations and the exploration of associated factors. This aligns with the theory’s focus on individual variability in stress responses. Therefore, we propose H2: Distinct FCR patterns in breast cancer patients can be identified using LPA.

**Figure 1:**
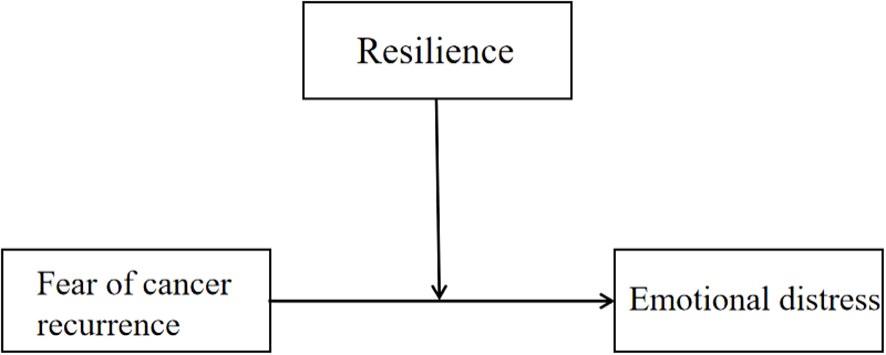
The hypothesized model.

Resilience, defined as the ability to adapt positively to adversity, plays a crucial role in moderating the impact of stressors on emotional outcomes (36). According to Stress and Coping Theory, resilience can influence both cognitive appraisal and coping strategies, thereby buffering the negative effects of stressors like FCR on emotional distress (31, 32). Resilient individuals are more likely to appraise stressors as manageable and employ adaptive coping strategies, such as seeking social support or reframing challenges positively (37, 38). Therefore, we propose H3: Resilience moderates the relationship between LPA-based FCR patterns and emotional distress in breast cancer patients. The proposed hypothetical model is illustrated in **Figure 1**.

## 3 Methods

### 3.1 Design and participants

In the period from May to December 2023, a mass of 398 breast cancer patients were recruited from three tertiary hospitals located in Guangdong Province for participation in the Be Resilient to Breast Cancer program (BRBC, a longitudinal project) (39–46). The study achieved a high response rate of 94.2%, with 375 participants successfully completing the survey. Initially, researchers provided a brief introduction regarding the study’s purpose and significance in a quiet and private setting. Participants who consented to take part were inquired to finish an informed agreement document, followed by a questionnaire. In instances where participants encountered difficulties in understanding the material, researchers offered clarifications and assistance in completing the questionnaire. The inclusion criteria for participation were as follows: (1) voluntary participation, (2) females older than 18 years, (3) Individuals who demonstrate a sufficient level of understanding, as assessed by the ability to comprehend the study purpose, procedures, and informed consent documents, and who can communicate effectively in Chinese, and (4) patients diagnosed for the first time after pathologic examination. The exclusion criteria included: Patients with concurrent malignant tumors in other organs or severe chronic diseases.

### 3.2 Sample size

To guarantee the reliability and precision of subgroup outcomes, LPA necessitates a smallest sample volume of 300 (47). Therefore, the sample size of 375 participants employed in the present current indicates a robust level of statistical power.

### 3.3 Instrument

#### 3.3.1 Demographic variables

Based on our group’s existing research (48–51) and clinical experience, we categorized the demographic variables into two distinct groups: (1) sociodemographic characteristics, which encompass age, education level, employment status, marital status, and financial burden, and (2) disease-related characteristics, which include menopausal status, duration of diagnosis, disease stage, molecular classification, and family history of cancer.

#### 3.3.2 Measurement of FCR

Fear of Progression Questionnaire-Short Form (FoP-Q-SF) was compiled by (52) by simplifying the FoP-Q scale. The Chinese adaptation of this instrument was translated by Qiyun Wu (53) and validated in Chinese patients with hepatocellular carcinoma, followed by a reliability test in breast cancer by Jianping Cai (54). It is a unidimensional scale containing 12 items on a Likert-5, with a total score ranging from 12 to 60. A mark of 34 or higher is typically regarded as the threshold indicative of FCR dysregulation. The research reported a Cronbach’s alpha coefficient of 0.923.

#### 3.3.3 Measurement of resilience

The 10-item Connor-Davidson Resilience Scale (CD-RISC-10) was extracted from CD-RISC-25 by (55), translated into Chinese and validated by Zengjie Ye (56, 57). The scale is a one-dimensional 10-item scale with a Likert-5 scale, with scores ranging from 0 to 40 on a scale of 0-4, representing “never” to “always”. The instrument demonstrates adequate psychometric characteristics and is deemed appropriate for assessing resilience levels among oncology sufferers (58). The Cronbach’s alpha was 0.940.

#### 3.3.4 Measurement of emotional distress

The Hospital Anxiety and Depression Scale (HADS) was developed by Zigmond and Snaith in 1983 (59), is a tool designed to screen hospital inpatients for emotional distress. The scale consists of 14 items divided into two subscales: the Anxiety Subscale (HADS-A) and the Depression Subscale (HADS-D), each comprising 7 items to assess anxiety and depressed mood, respectively. Each item is scored on a 4-point Likert scale (ranging from 0 to 3), with total scores for each subscale ranging from 0 to 21. Higher scores indicate more severe emotional distress. The HADS has been broadly utilized in cancer survivors (60, 61). The Cronbach’s alpha coefficient in this study was 0.854.

### 3.4 Data analysis

First, demographic variables were reported as mean ± standard deviation for continuous variables (e.g., age) and frequencies and proportions (%) for categorical variables (e.g. education level). The normality of continuous variables was assessed using the Shapiro-Wilk test. Parametric tests were used for variables that met the assumption of normality, while non-parametric tests were applied for variables that did not. A univariate analysis was conducted to explore potential factors associated with the onset of emotional distress. Subsequently, Pearson’s correlation analysis was performed to examine the relationships among FCR, resilience, and emotional distress, as the data met the assumptions of normality and linearity. Third, LPA was utilized to recognize potential subgroups of breast cancer patients exhibiting FCR, utilizing the FoP-Q-SF scores. The analysis began with a one-class model and continued until further improvements in fit indices were no longer statistically significant. The fitting indices employed to verify the ideal count of profiles included the Akaike information criterion (AIC), Bayesian information criterion (BIC), and sample-size-adjusted BIC (aBIC). Additionally, significant values from the Lo–Mendell–Rubin likelihood ratio test (LMR) and the bootstrap likelihood ratio test (BLRT) indicated that the model with K categories was preferred to the model with K-1 categories (62, 63). Univariate logistic regression analyses were then conducted to ascertain factors linked with the various profiles according to LPA, with outcomes illustrated through forest plots. A Bayesian Factor (64) was employed to assess the differences in HADS scores across various LPA profiles. In the fourth step, Harman’s single-factor test was applied to examine the potential presence of common method bias (65). A single factor explaining less than 40% of the variance was considered indicative of no significant common method bias. Fifth, analyses were performed according to the two dimensions of the HADS to examine the moderating role of resilience between LPA-based FCR and anxiety and depression respectively, through the PROCESS macro (Model 1) (66). Johnson-Neyman plots were drawn to describe the interactions (67). Statistical significance was set at a threshold of α=0.05. Data for this study were processed using SPSS 25.0 (Armonk, NY: IBM Corp), Mplus (8.3), and JASP (0.18.1), R (4.4.2).

## 4 Results

### 4.1 Demographic variables

49.1% of the patients had a middle school education or less. 35.2% of the patients were still in service after illness. More than half of the patients were menopausal (50.4%). 42.4% of the patients belonged to the Luminal B type. Significant differences were identified between emotional distress based on financial burden (*P* = 0.002). **Table 1** presents comprehensive information.

**Table 1:** Demographic characteristics among breast cancer patients.

Variables	Outcome variable: Emotional distress	
	M ± SD/N (%)	*P* value
**Age**	49.36 ± 10.746	—
**Education**		0.315
Middle school and below	184 (49.1%)	
High school	85 (22.7%)	
University or above	106 (28.3%)	
**Occupational status**		0.216
Employed	132 (35.2%)	
Unemployed	136 (36.3%)	
Retirement	107 (28.5%)	
**Marital status**		0.790
Unmarried/divorced/widowed	70 (18.7%)	
Married	305 (81.3%)	
**Menopausal or not**		0.065
Yes	189 (50.4%)	
No	186 (49.6%)	
**Financial burden**		**0.002**
No burden	53 (14.1%)	
Mild-moderate	178 (47.5%)	
Heavy	144 (38.4%)	
**Diagnosis time**		0.652
<3 months	117 (31.2%)	
3-6 months	81 (21.6%)	
6 months -1 year	127 (33.9%)	
>1 years	50 (13.3%)	
**Molecular typing**		0.542
Luminal A	55 (14.7%)	
Luminal B	159 (42.4%)	
HER-2 overexpression	106 (28.3%)	
Triple negative	37 (9.9%)	
unknown	18 (4.8%)	
**Stage of disease**		0.486
0+I	145 (38.7%)	
II	137 (36.5%)	
III+IV	93 (24.8%)	
**Family history of cancer**		0.112
Yes	54 (14.4%)	
No	321 (85.6%)	

Bolded values indicate statistically significant results (p < 0.05).

### 4.2 Pearson correlation analysis of FCR, resilience and emotional distress

**Figure 2:**
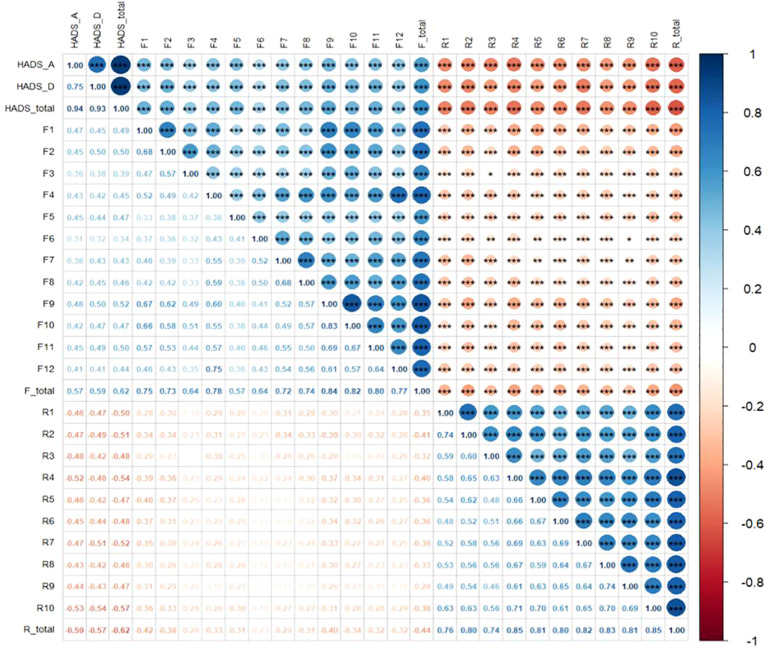
Pearson correlation heatmap among fear of cancer recurrence, resilience, and emotional distress. (1) F, Fear of cancer recurrence; R, Resilience; HADS-A, Anxiety subscale; HADS-D, Depression subscale. (2) Significant levels are shown at the top right of the figure, ***<0.001, **<0.01, *<0.05.

Harman’s one-factor model displayed that the primary element answered for 27.97% of the whole variance, implying that the potential impact of shared method bias was considered minimal for this research. As well, a noteworthy negative connection was discovered between FCR and resilience (r = −0.441, *P* < 0.001), while a notable positive bond was identified between FCR and emotional distress (r = 0.619, *P* < 0.001). Additionally, a significant negative tie was established between resilience and emotional distress (r = −0.618, *P* < 0.001). Further details are exhibited in **Figure 2**.

### 4.3 LPA of FCR

**Figure 3:**
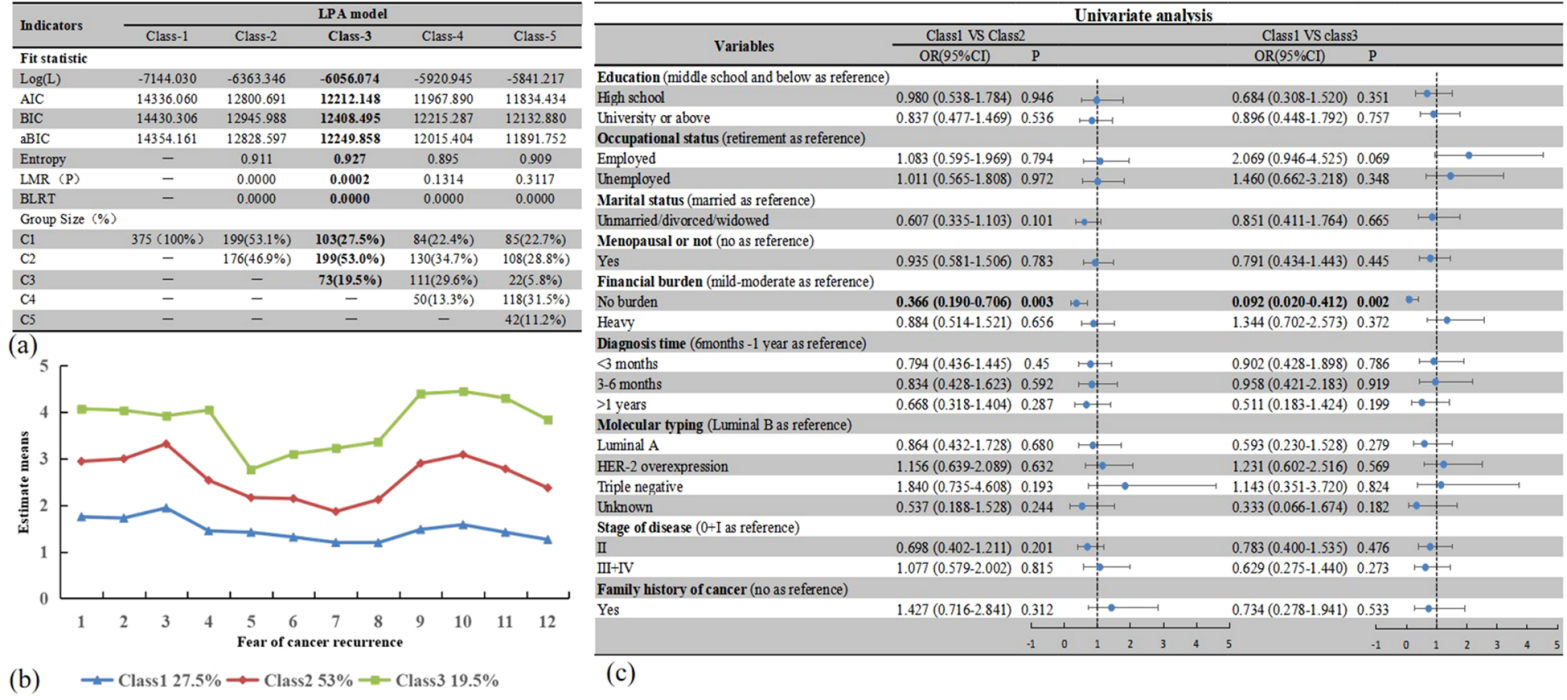
Latent profile analysis models and logistic regression results. **(a)** Fitting index and group size of LPA models; **(b)** Parameters for the final 3-class; **(c)** logistic regression results based on LPA. Bold values emphasize the selection of the best profile, or indicate statistically significant results (*P*<0.05).

A 3-class model was chosen over a 2-class model for the following reasons: (1) The values of various fitting indices such as AIC, BIC, and aBIC were relatively low; (2) The entropy value is close to 1 and (3) LMR and BLRT were notable (P < 0.05). **Figure 3a** shows the match indicators of the different models. The three profiles were termed low FCR (27.5%, N = 103, Class1), middle FCR (53%, N = 199, Class2), and high FCR (19.5%, N = 73, Class3). **Figure 3b** presents details about LPA-based profiles. Univariate logistic regression demonstrated financial burden differed across the three profiles. **Figure 3c** describes the other details.

### 4.4 LPA-based difference in HADS scores

**Figure 4:**
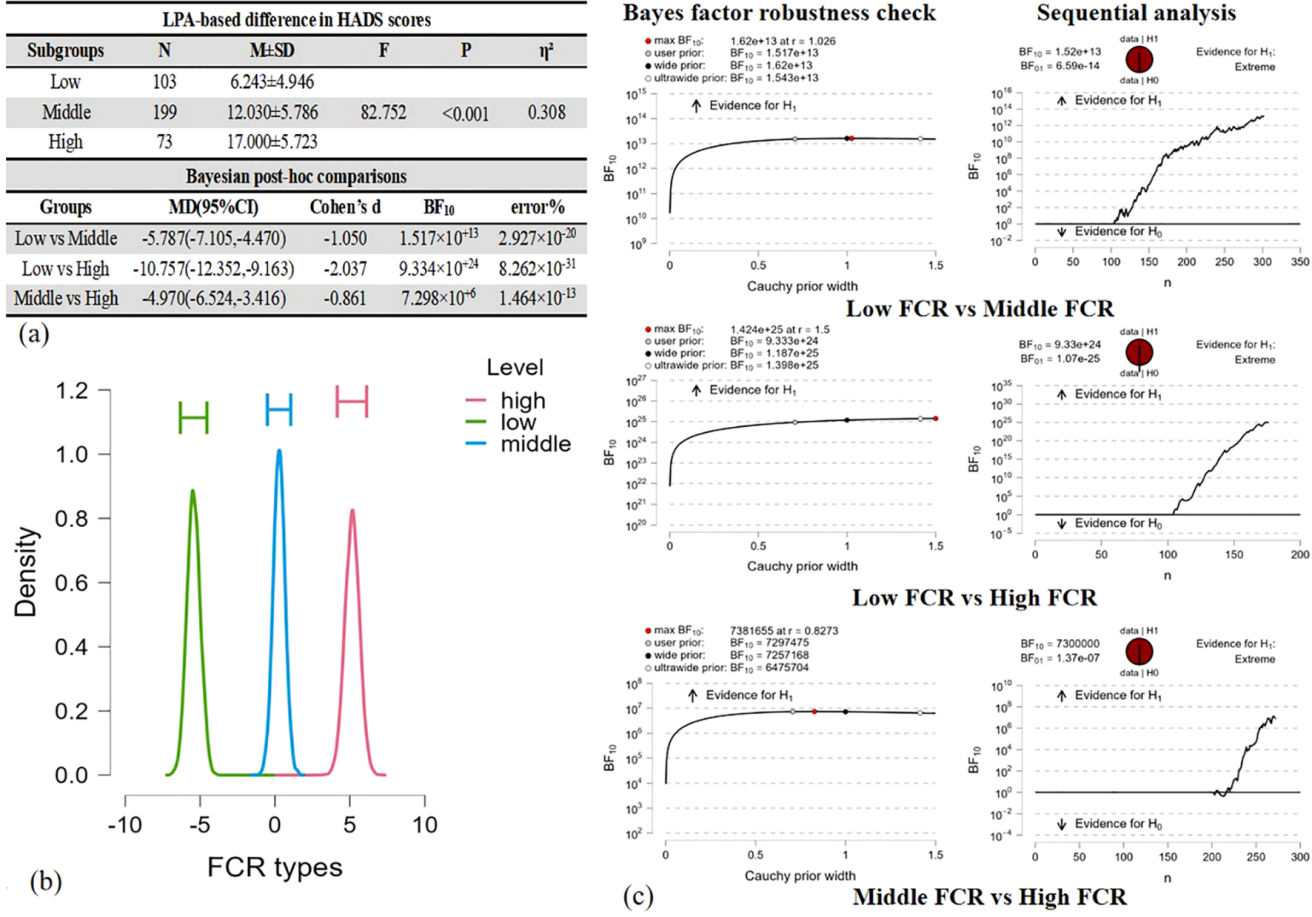
ANOVA comparison of HADS scores across LPA-based groups and *post-hoc* comparison by Bayesian factor analysis. **(a)** LPA-based differences in HADS scores were estimated by Baysian Factor. **(b)** ANOVA model averaged posterior distribution. **(c)** Inferential plots for Bayesian factor analysis.

Significant group distinctions in HADS scores (F = 82.752, *P* < 0.001) were noted between the “low FCR” and “middle FCR” groups (BF10 = 1.517×10^+13^, d = −1.050), between the “low FCR” and “high FCR” groups (BF10 = 9.334×10^+24^, d = −2.037) and between the “middle FCR” and “high FCR” groups (BF10 = 7.298×10^+6^, d = −0.861). **Figure 4** presents detailed information.

### 4.5 Moderation analysis of resilience based on LPA

**Figure 5:**
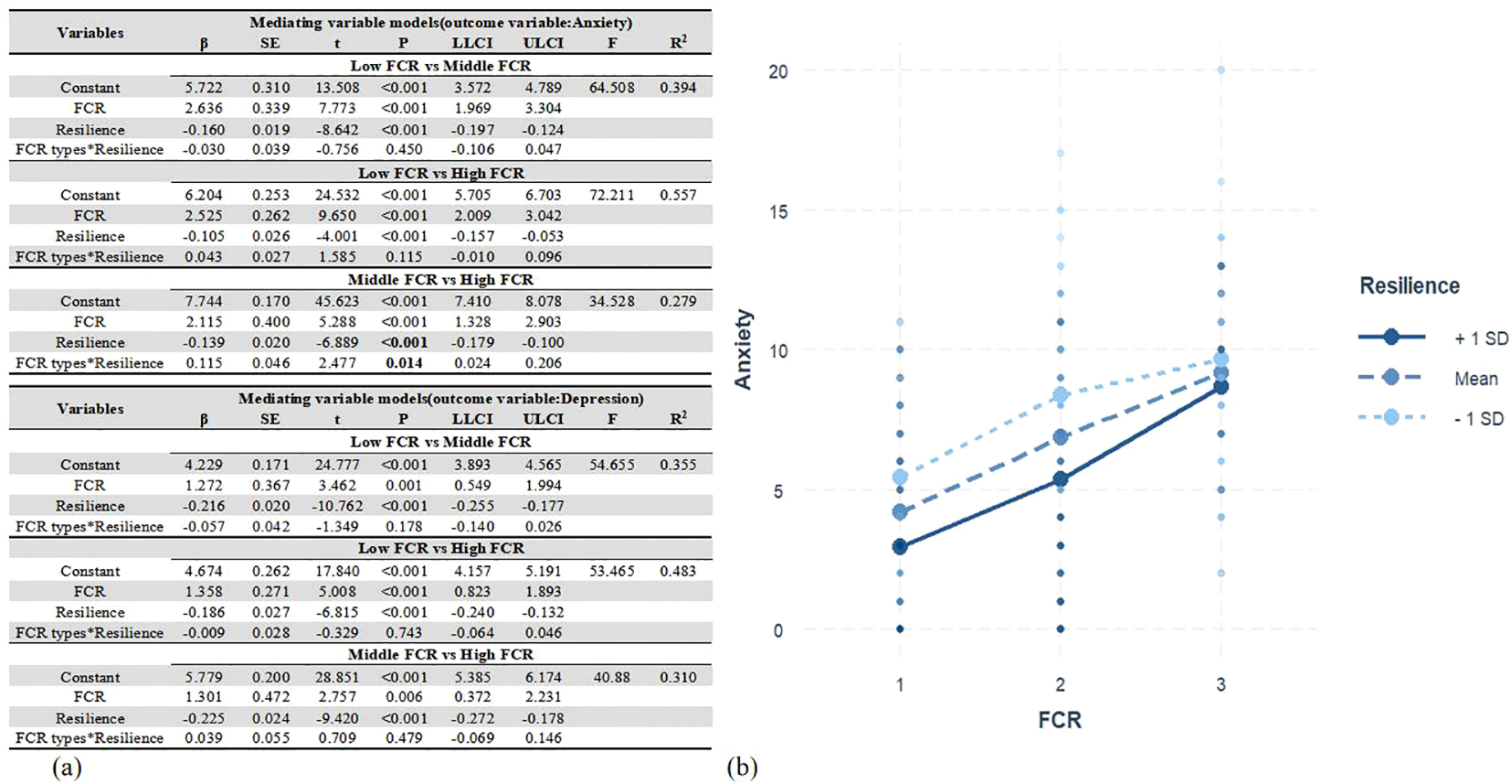
The moderating effect of resilience between LPA-based FCR and emotional distress. **(a)** Moderating role of resilience in the association between FCR and anxiety and depression. **(b)** Plot of interaction between FCR and resilience on anxiety.

The moderation analyses indicated that the interactions between types of FCR (Middle FCR vs High FCR) and resilience were significantly correlated with anxiety (B = 0.115, SE = 0.046, *P* = 0.014). Conversely, no notable moderation results were observed between the Low FCR and Middle FCR groups, nor between the Low FCR and High FCR groups across varying levels and resilience (B = −0.030, SE = 0.039, *P* = 0.450, and B = 0.043, SE = 0.027, *P* = 0.115, separately). Furthermore, the interactions between FCR types and resilience did not demonstrate a significant association with depression (*P* > 0.05). Additional information can be discerned from **Figure 5**.

## 5 Discussion

This study highlights the heterogeneity of FCR in breast neoplasm patients, categorizing it into low, middle, and high levels. Results show a significant positive correlation between FCR and emotional distress, suggesting that higher FCR levels are linked to greater emotional burden. Additionally, resilience appears to mitigate the impact of FCR on anxiety but not on depression.

First, the current investigation revealed a positive correlation between FCR and emotional distress among breast neoplasm patients, aligning with the findings of a meta-analysis encompassing 31,740 patients (68). Previous research has established that FCR, anxiety, and depression are co-morbid conditions that mutually exacerbate one another’s effects during the treatment and recovery phases (69). Moreover, this study identified a significant association between financial burden and emotional distress, corroborating the outcomes of earlier articles (70). The substantial payments related to therapy and the protracted rehabilitation process contribute to inactivity and financial strain, which represent a considerable burden for families. To sum up, we, as clinical workers, should popularize the relevant professional knowledge of breast cancer for patients, so as to reduce unnecessary worries; and use music therapy (71) and exercise therapy (72) to improve the negative emotions of patients in the course of treatment. The government should increase its investment in medical insurance and improve the coverage and reimbursement rate of medical insurance to reduce the financial burden of patients (73).

Second, consistent with our H2 and previous research findings (74, 75), the research sample of breast cancer patients was classified into three distinct profiles based on their levels of FCR (low, middle, and high). Specifically, Profile 1 members (low FCR) exhibited minimal FCR, which may be attributed to effective coping mechanisms or strong social support systems (76). These patients typically experience lower levels of emotional distress and better psychological well-being (13). However, even in this group, periodic monitoring and mild supportive care are recommended to maintain their resilience and prevent potential escalation of FCR. Profile 2 members (middle FCR) represented the majority of the sample (53%). These patients are more susceptible to stress-inducing events during treatment and recovery, making them prime candidates for interventions such as mindfulness meditation exercises (77), organized group discussions, and team-based psychological support. These interventions aim to enhance emotional regulation and reduce stress-related symptoms (78). Profile 3 members (high FCR) demonstrated severe FCR, which is associated with maladaptive coping strategies and heightened psychological distress (79). For these patients, structured counseling interventions delivered by trained oncology nurses (80), combined with personalized psychological therapy and continuous monitoring, are essential to mitigate their distress and improve their quality of life (81). The differences between these profiles may stem from various factors, including individual coping styles, availability of social support, and prior experiences with cancer treatment (13). For instance, patients with low FCR may have stronger social networks or better access to psychological resources, while those with high FCR may face additional stressors, such as financial difficulties (82) or comorbid mental health conditions (20). Therefore, future studies should include more diverse populations and consider the role of emotional and instrumental support systems (83) in alleviating FCR and promoting healthier psychological and physical conditions among breast cancer patients.

Third, this study confirms that resilience serves as a significant moderating factor between FCR and anxiety, but not between FCR and depression. These findings have important implications for clinical practice. Previous studies have shown that approximately one-third of breast cancer patients continue to experience significant FCR within five years following their diagnosis (84). Persistent and intense fear can deplete patients’ internal psychological resources and exacerbate their emotional burden, leading to increased anxiety and reduced quality of life (85). Resilience, as a protective psychological factor, has been demonstrated to have a notable negative correlation with FCR (23), as well as with anxiety and depression (86). Patients with higher resilience are better equipped to mitigate the adverse effects of FCR, thereby reducing anxiety. The lack of a significant moderating effect of resilience between FCR and depression may be attributed to the relatively lower reported levels of depression in our sample, which warrants further investigation. In clinical practice, these findings underscore the importance of incorporating resilience-building strategies into psychological interventions for breast cancer patients. For example, cognitive-behavioral therapy (CBT) (78) and mindfulness-based interventions could be tailored to enhance patients’ resilience, helping them to better cope with FCR and reduce anxiety. Overall, breast cancer survivors can benefit from actively developing psychological resilience, which enables them to effectively regulate negative emotions and face life challenges with optimism (87, 88).

## 6 Limitations

The current research exhibits various constraints. Firstly, the sample utilized is derived exclusively from Guangdong Province, which may limit its representativeness. Subsequently, the outcomes may not suitable to patients in extra zones. Secondly, as this research employs a cross-sectional design, it does not facilitate an examination of causal relationships. Hence, the sample should be expanded with longitudinal studies to further validate the correlations in the future study. What’s more, future investigations should consider exploring the moderating result of resilience on the bond between FCR and various symptoms associated with emotional distress.

## 7 Conclusion

Heterogeneity exists in FCR among individuals diagnosed with breast cancer. Resilience plays a significant moderating role between FCR and anxiety. It is imperative for clinicians to focus on patients exhibiting middle to high levels of FCR, to incorporate resilience-based psychological interventions, and to assist those with elevated FCR in mitigating emotional distress while fostering confidence in their ability to navigate their cancer journey.

## Data Availability

The original contributions presented in the study are included in the article/supplementary material. Further inquiries can be directed to the corresponding author/s.
